# Efficient Security Mechanisms for mHealth Applications Using Wireless Body Sensor Networks

**DOI:** 10.3390/s120912606

**Published:** 2012-09-17

**Authors:** Prasan Kumar Sahoo

**Affiliations:** Department of Computer Science and Information Engineering, Chang Gung University, Kwei-Shan 33302, Taiwan; E-Mail: pksahoo@mail.cgu.edu.tw; Tel.: +886-3-211-8800; Fax: +886-3-211-8668

**Keywords:** wireless body sensor networks, mHealth, security, authentication, confidentiality

## Abstract

Recent technological advances in wireless communications and physiological sensing allow miniature, lightweight, ultra-low power, intelligent monitoring devices, which can be integrated into a Wireless Body Sensor Network (WBSN) for health monitoring. Physiological signals of humans such as heartbeats, temperature and pulse can be monitored from a distant location using tiny biomedical wireless sensors. Hence, it is highly essential to combine the ubiquitous computing with mobile health technology using wireless sensors and smart phones to monitor the well-being of chronic patients such as cardiac, Parkinson and epilepsy patients. Since physiological data of a patient are highly sensitive, maintaining its confidentiality is highly essential. Hence, security is a vital research issue in mobile health (mHealth) applications, especially if a patient has an embarrassing disease. In this paper a three tier security architecture for the mHealth application is proposed, in which light weight data confidentiality and authentication protocols are proposed to maintain the privacy of a patient. Moreover, considering the energy and hardware constraints of the wireless body sensors, low complexity data confidential and authentication schemes are designed. Performance evaluation of the proposed architecture shows that they can satisfy the energy and hardware limitations of the sensors and still can maintain the secure fabrics of the wireless body sensor networks. Besides, the proposed schemes can outperform in terms of energy consumption, memory usage and computation time over standard key establishment security scheme.

## 1. Introduction

Wireless communications fundamentally modernizes our life style and changes the way of health care services. There is growing interest on prevention and early detection of disease or optimal maintenance of chronic conditions to augment existing health care systems. The advent of wireless sensor networking (WSN) can play vital role to design efficient Mobile Health (mHealth) systems to react to the crisis and to manage the illness. Moreover, the advances in nano technology, mobile networks, pervasive computing, wearable systems, and drug delivery approaches are transforming the health care sector. Combination of these innovative technologies can be used in various health care practices, acute care, and preventative health. These developments not only have had a significant impact on current e-health [1] and telemedical systems, but they also are leading to the creation of a new generation of Mobile Health (mHealth) [2] systems with a convergence of devices, technologies, and networks at the forefront of the innovation. Wireless Body Sensor Network (WBSN) can provide long-term health monitoring without disturbing the privacy of a patient. Important applications of WBSN comprises vital sign monitoring, home care monitoring, clinical monitoring, and sports-person health status monitoring [3].

Some of the most compelling benefits of mobile technologies are in the areas of disease prevention, chronic disease management and improving health care delivery. mHealth is defined as “mobile computing, medical sensor, and communications technologies for healthcare”. The use of the mHealth terminology relates to applications and systems such as telemedicine, telehealth, and biomedical sensing system. Due to high cell phone usage, mobile devices have become necessary tools in our daily life, and it is time to make use of mobile for providing patient service. The process of the mHealth is conducted by mobile technology, and its primary function is to help physicians and hospital administrators manage individual patients in a systematic fashion. Since cell phones are so popular all over the world, it is time to launch mobile healthcare service for patients, physicians and hospital administrators. Better service quality in medical care and health care can be fulfilled by mobile technology. This mHealth service not only improves the quality of health care services, but also improves patient relationships for health care providers. If the mHealth enlarges its service domain, it has the potential to be a multi-functional health management agent in the future.

One of the most promising applications of sensor networks is for human health monitoring. There is a critical need for more cost efficient solutions for supervision/monitoring physiological signals of chronic heart patients using wireless body sensor networks even if they are at home, moving outside or driving vehicles. A number of tiny wireless body sensors, strategically placed on the human body, create a wireless body area network that can monitor various vital signs, providing real-time feedback to the user and medical personnel. The wireless body sensor networks promise to revolutionize health monitoring. Advanced sensors combined with wireless communication can reduce costs, improve monitoring, and better life quality for the patient. The benefit of using wireless sensor technology in health care can be divided into two areas. One area is the use of new technological solutions for individually based, multi-parameter monitoring at home. Patients with chronic diseases, as well as a constantly growing number of seniors, will prof t on treatment and medical monitoring in their own environment (e.g., at work or at home). These monitoring systems are linked to individuals rather than places. Almost unlimited freedom of movement implies use of wireless and even implanted sensors that will greatly enhance home monitoring and follow-up. The second area of benefit lies within increasing the efficiency of treatments at hospitals. The cost of continuous monitoring and surveillance is already high and is growing dramatically. This goes for both prior to treatment monitoring, and internally at the hospital, as well as post-monitoring.

The wireless body sensors of today are mostly based on hard wiring, in addition to being based on proprietary solutions. There are several forms of body sensors available in the market, such as piezo-electrical materials for pressure measurements, infrared sensors for body temperature estimation, and optoelectronic sensors that monitor heart rate and blood pressure. These sensors are being embedded into wearable items and accessories that can be carried easily. With the continual improvements to the sensors and the miniaturization of computing devices, these wearable devices for monitoring, diagnosing, and treating illnesses are becoming more readily available and are a key technology in helping the transition to more proactive and affordable health care. These wearable wireless body sensors allow an individual to closely monitor changes in some ones vital signs and provide feedback to maintain an optimal health status. If integrated into a telemedical system, these systems can even alert medical personnel when life-threatening changes occur. Besides, multi-parameter analysis produces new data that can enhance information quality.

The implementation of more flexible wireless technology will lead to reduced hospitalization time due to more rapid mobilization, as well as improved documentation by stored, digitalized signals. The result will be enhanced decision making for diagnostics, observation and patient treatment. Hence, use of mHealth technology has social and financial implications along with providing special care to the aged adults remotely. However, wireless body sensor networks have a few inherent limitations. e.g., limited hardware, limited transmission range, and large scale network system and the traditional protocols cannot use in WBSNs. The sensors in WBSNs are equipped with special sensing modules such as an electrocardiogram, pressure or temperature sensor. These sensors are fitted at different parts of a patient in form of a smart suit and transmit data to a mini gateway node located within the same smart suit. This mini gateway node is responsible for organizing and transmitting data to a powerful sensor that acts as a network connector, which forwards the data to the final destination with higher storage and processing capabilities.

Sensing devices in WBSNs must be capable of routing packets on behalf of other devices to the base station or sink and finally to the end users. The end users receive the data in a multihop infrastructure-less architecture through the sink. The sink enables communication between the host application and sensors. Wireless body sensors mainly use broadcast communication by which on one hand they affect the trust assumptions, and on the other they minimize energy usage. Each node can forward a message towards a sink, recognize packets addressed to it, and handle message broadcasts. The base station accesses individual nodes using source routing. The task manager in WBSNs is responsible for configuration of the network, scheduling, communication between devices, management of routing tables and monitoring and reporting the network. Other than a task manager, a security manager is responsible for the generation, storage, and management of keys.

Recently, security is becoming an important research topic in the WBSNs based mHealth application, as many applications of mHealth have urgent need to protect confidential data. These applications range from the indoor applications like smart home to health monitoring in a hospital. Since wireless sensors rely on broadcasting, any adversary can eavesdrop on traffic, inject new messages, and replay old messages. The adversary may use its own formula of attacking and induce the network to accept them as legitimate nodes. Falsification of original data, extraction of private sensed data, hacking of collected network readings and denial of service are also certain possible threats to the security and privacy of the sensor networks.

Sensor nodes may not be tamper resistant and if an adversary compromises a node, it can extract all key material, data and code stored on that node. While tamper resistance might be a viable defense for physical node compromise for some networks, we do not see it as a general purpose solution. Extremely effective tamper resistance tends to add significant per-unit cost, whereas sensors are intended to be very inexpensive. Though hardware and software improvements may address many of such security issues, development of new supporting technologies and security principles are challenging research issues in WBSNs. Similarly to conventional networks, most applications of wireless body sensor networks require protection against eavesdropping, injection, or modification of disseminated data packets. Cryptography is the standard method of defence against such attacks, but brings a number of trade-offs into play. Hence, several issues are needed to consider in WBSNs to construct an efficient and robust network. Since wireless body sensors have limited computation capability and limited power supply, low complexity algorithms and power saving schemes respectively should be considered to plan efficient security solutions.

In this paper, lightweight security protocols for the mHealth application using wireless body sensor networks are proposed that requires limited memory usage and computation time and therefore can minimize the energy consumption. The rest of the paper is organized as follows. Related works of wireless body sensor networks security issues are discussed in Section 2. A three tier architecture of wireless body sensor networks for the mobile health system is proposed in Section 3. Corresponding system model of the proposed WBSNs is designed in Section 4 and light weight security schemes are illustrated in Section 5. Performance evaluation of our security schemes is given in Section 6 and concluding remarks are made in Section 7.

## 2. Related Work

Normally, wireless sensor network is enabled with Link-layer security suites and most sensors have a built-in advanced encryption system (AES) for cryptographic operations. However, the major drawback of the built-in cryptosystem is that it does not offer AES-based decryption, due to which the encrypted data cannot be accessed by an intermediary node during communication. Besides, hardware based built-in cryptosystem makes the application highly platform dependent. A wide-area mobile patient monitoring system named as MobiCare [4] is designed to monitor the physiological status of patients. In this project, the MobiCare client comprises a wristwatch and the back-end is the MobiCare server. Though authors propose a wireless transport layer security protocol for data integrity and authentication, security and privacy is still not implemented in the proposal. Though a public-key cryptosystem can have many advantages, such as strong security, scalability, and memory efficiency, it is computationally expensive for power constraint wireless body sensors. Elliptic curve cryptographic [5,6] schemes have been found suitable for the resource constrained networks, but practical signature generation and verification are still expensive in term of the time complexity in real-time implementation.

Since communication in WBSN is based on broadcasting, there are every possibilities that attacker can eavesdrop the message and reply it. In a sinkhole attack, adversary tries to attract nearly all the traffic from a particular area and creates a sinkhole in the network. It causes the routing algorithm to attract other nodes to send their data through it, manipulates the data and then sends them to the sink. In the Sybil attack [7], a single node presents multiple identities in the network to put other nodes in trouble. In a multi-hop packet forwarding environment [8,9], data packets are expected to be forwarded to the base station or remote server via multi-hop routing. By using selective forwarding attack, malicious nodes refuse to forward certain data such as ECG, heart beat and temperature, *etc.* and simply drop them, so that they cannot be broadcast further. This threat can be stronger if the attacker is explicitly included in the routing path. In the Wormhole attack [10], the adversary tunnels messages received in one part of the network over a low latency link and replays them in a different part. It creates sinkhole in the network and the shortest route among the nodes to the base station to intercept the message. This attack may be used in combination with selective forwarding or eavesdropping. Another potential attack is the hello food attack and it is similar to the broadcast wormholes attack. In this attack, it uses a single hop broadcast to send a message to a number of receivers.

Several symmetric algorithms [11,12] are proposed for broadcast authentications, but such algorithms are not suitable for WBSNs due to high communication overhead per packet. The security protocols for sensor networks (SPINS) [13] such as SNEP and *μ*-TESLA have been proposed for the resource constrained wireless sensor networks (WSNs). Several key exchange, distribution and management protocols [14,15] are also proposed for the pre- or post-deployed sensor nodes. In LEAP [16], a key management protocol for the sensor networks that support the in-network processing is proposed. This protocol supports the establishment of four types of keys for each sensor nodes and are used for establishing and updating the keys and simultaneously minimizes the involvement of the base station. An adaptive key selection scheme [17] and its corresponding algorithm is proposed for the multiple deployment in sensor networks. Though they use three different types of asymmetric keys for encryption, there will be communication and computational overhead to the cluster head nodes. In [18], authors use the historical data of the target region to generate a hierarchical key structure for managing the group keys. However, it incurs storage problem for the memory constraint sensor nodes.

The key management and key establishment schemes for wireless sensor networks are extensively studied in [19–22]. In [15], a scalable, power efficient secure protocol is proposed for the WSNs. This protocol allows each sensor node to share two types of keys, e.g., a master key shared with the base station and an explicit key between individual neighboring nodes to exchange the secure information. It is observed that some of key distribution protocols propose the key assignments at the pre-deployment phase, whereas some other protocols propose the key distribution and management at the post-deployment phase. However, sharing of pairwise keys at the pre-deployment phase is not suitable for the power and memory constraint sensor nodes. Similarly, key establishment among sensors at the post-deployment phase is not feasible as sensors are normally deployed randomly. A link layer group communication scheme [23] for the wireless sensor networks is proposed to achieve security of the node-to-node communication. Though the scheme is independent of the key management and distribution architecture, there is no mention about data confidentiality, which is highly essential for the WSNs deployed on the battle field and similar security applications.

The mutual user authentication scheme or session-key agreement between the sensors and sink is proposed [24–26] to authenticate any user to access to the WSNs. A user authentication scheme [24] is proposed, which is executed by the user and the coordinator of the WSN. The password is memorized by the user and a secret key is saved in each device to authenticate the identity of any sender. The authors in [26] uses a strong password-based authentication schemes for the remote user authentication. In these protocols there is no analysis how data confidentiality is maintained. Other than the data confidentiality and authentication, data freshness is an important factor in healthcare applications. In mHealth applications, physiological signs of a patient should be fresh and adversary should not replay the old messages to confuse the doctors and care givers. Normally there are two types of data freshness [27] considered in wireless security. The weak freshness does not carry time-delay information and gives partial message ordering, whereas a total order on a request-response pair that allows the delay estimation is considered in strong data freshness.

Authors in [28] summarizes these types of threats seen in mHealth applications. They are the misuse of patient identities, unauthorized access or modification or disclosure of personal health information. In each category, the adversary could be the patient, insiders such as authorized personal health record users, or organization, and outsiders such as third parties. Though authors discuss all possible cases of threats, they do not propose any security mechanism as the solution to those threats. A secure same symptom-based handshake scheme [29] is proposed for the mHealth application in which each patient is granted with a pseudo-ID and a private key corresponding to his symptom. Patients can use their private keys to make mutual authentication if they have the same symptom. However, the proposed scheme is for mHealthcare social network built upon wireless body sensor network and the security mechanisms are not meant for the data authentication and confidentiality that are exchanged among the body sensors. In wireless body sensor networks, normally sensors sense different parameters of the body and transmit it either to the physician or the hospital server. During the data transmission, the data could be hacked as the adversary can capture the physiological data from the wireless channels, and can change it. Besides, the gateway nodes in wireless communication are normally unguarded, which can provide unrestricted access to an attacker. Hence, in this case an illegal gateway node can act as a real node to transmit data to other nodes of the the network.

An adaptive fault-tolerant communication scheme for wireless body sensor networks is proposed in [30], in which channel bandwidth is reserved for specific sensors who transmits critical human physiological data. Thus, a reliable data transmission mechanism is maintained by the body sensor networks based on the external environment. A secure health monitoring environment is created for a patient by using medical wireless sensor networks as discussed in [31]. The proposed environment can detect ECG signals wirelessly within the patient body and can provide reliable data transmission with minimum consumption of power using TelosB technology. Besides, the proposed schemes can achieve the data confidentiality, authenticity, and integrity of the patient's data at low computation and communication cost. Since mHealth applications are not limited to monitoring a patient's physiological data, strict confidentiality should be maintained to secure the privacy of a patient by sharing healthcare data with doctors, health insurance companies such as healthcare data access, and electronic health records. Hence, security should be maintained in mHealth applications from the social and ethical point of view. The authors in [32] discuss a detail survey of security and privacy issues in healthcare application using wireless body sensor networks. In their study, authors highlight some popular healthcare projects and discuss different aspects of security and privacy issues in wireless body sensor networks.

From the study of several latest literature, it is observed that most of the works analyze different aspects of securities in mHealth applications. However, to the best of our knowledge none of the work propose data confidentiality and authentication protocols for the wireless body sensor networks taking memory and computation as constraints, though several security mechanisms are proposed for the wireless sensor networks (WSN), which is different from the WBSN. In this paper, a light weight data authentication and data confidentiality scheme for the wireless body sensor networks is proposed for the realization of the mobile health applications and to minimize the computation and memory usage of the proposed algorithms. The motivations and main contributions of our proposed scheme are given as follows.

### 2.1. Motivations

So far, the security schemes proposed for the WBSNs are mainly at the architectural level and are limited to key establishment and authentication. The main motivations by several researchers for designing security protocols for the WBSNs are to develop light weight security schemes in terms of computation and memory usage so that the network lifetime could be improved. Though the proposed protocols are more or less suitable to be used in wireless body sensor networks, most of them are not feasible in real implementation due to power and memory constraints of the sensors. Most of the previously proposed key distribution protocols are not suitable for the WBSNs due to large memory requirement, key establishment timings and more precisely as the body sensors are not deployed randomly. The symmetric key sharing among the nodes of the network is an important design issue for the security protocols. Though this key sharing approach has the lowest storage costs and power efficient, there are obvious security disadvantages such as the compromise of a single node will reveal the global key.

The sharing of pairwise keys between two nodes is more ideal since the compromise of a node does not reveal any keys. However, in this approach each node requires a unique key and keying relationship needs to be established after the network is deployed. Previously proposed asymmetric key methods such as digital signatures for the authentication are impractical as long signatures incur high communication overhead of 50∼1,000 bytes per packet to create and verify the signature. Another design security issue in WBSNs is to maximize the lifetime of body sensors. Hence, computation and operations of nodes during possible security verifications should be energy efficient and satisfy the hardware constraints. In order to minimize the number of shared keys, in this paper, we propose the light weight data confidentiality and authentication schemes without considering the key distribution or establishment among the nodes. A three tier network architecture along with light weight, low complexity data confidentiality and user authentication schemes are proposed for the WBSNs. The main contributions of our work can be summarized as follows:

- First of all, a three tier network architecture for the wireless body sensor networks (WBSNs) is proposed.
- Security and privacy challenges in WBSN are discussed and respective implementation feasibilities of currently available body sensors are analyzed.
- Based on the hardware constraints such as memory and computation capabilities of body sensors, a light weight security mechanism for the proposed three tiered WBSN network architecture is designed.
- In order to minimize the memory usage of nodes, key sharing with stronger security methods at the data communication level is developed such that the whole network requires only three types of keys and each sensor needs to store only one key.
- Light weight data confidentiality as well as authentication algorithms are designed for the body sensors as privacy of health related data of a patient should be maintained.
- A segmentation based data packet communication scheme is designed, where secret keys are embedded within those segmented packets for necessary verification of authentication and data confidentiality.

## 3. A Three Tier WBSN Architecture

**Figure 1.**
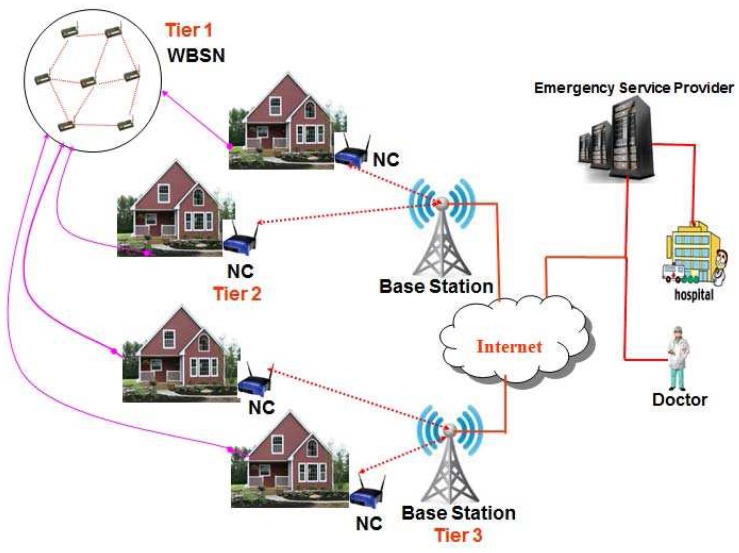
The proposed three tier Wireless Body Sensor Networks.

Let us consider a three tier heterogeneous wireless body sensor networks, which is mainly proposed for patient health monitoring in an indoor environment. Though the three tier WBSN architecture proposed in this paper is limited to the security mechanisms between wireless sensors with base station, ultimately the sensed data can be forwarded to an Emergency Service Provider (ESP) that comprises a medical server optimized to service hundreds or thousands of individual users, and encompasses a complex network of interconnected services, doctors and health care professionals as shown in Figure 1. A patient's physician can access the data remotely via Internet and examine the heart beat rate and blood pressure to ensure that the patient is responding to a given treatment for realization of a complete mHealth application.

**Figure 2.**
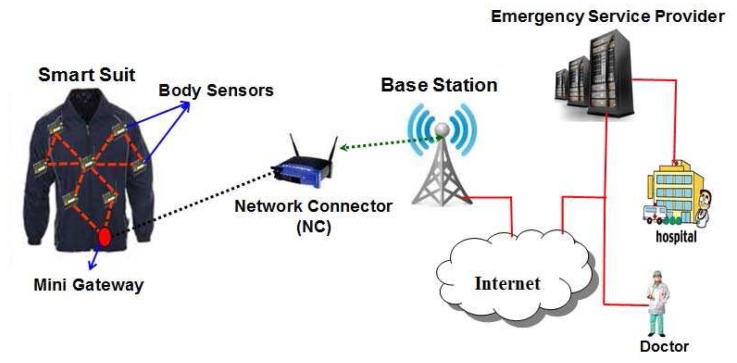
Communication architecture of three tier Wireless Body Sensor Networks for indoor mHealth applications.

Wearable systems for continuous health monitoring are a key technology in helping the transition to more proactive and affordable healthcare. They allow an individual to closely monitor changes in one's vital signs and provide feedback to help maintain an optimal health status. If integrated into a telemedical system, these systems can even alert medical personnel when life-threatening changes occur. Normally, an mHealth architecture can be divided into two segments, viz., indoor and outdoor environments. However, in this proposed network architecture, wireless body sensor networks for an indoor environment is considered as shown in Figure 1. As shown in Figure 1, it is assumed that a chronic patient who stays at home is fitted with several body sensors to monitor temperature, pulse rates, heart beats *etc.*, and is attributed as an indoor environment. The related body sensors can be fixed in a wearable suit, wristwatch, ring and sucks. It is assumed that each house has a powerful sensor to collect data from those body sensors and it acts as a network connector (NC). Thus there are several network connectors located in each house and they communicate with each other to transmit data to the nearest gateway node or base station. Considering the body sensors, network connectors and gateway nodes, a wireless body sensor network (WBSN) is formed. By using the WBSN, the collected data sensed by the body sensors is transmitted to an emergency service provider via internet as shown in Figure 2. Note that all physiological data of a patient can be transmitted to the mobile phones of the doctors or care givers from time to time for its analysis and to provide proper healthcare to a patient. The whole communication architecture can be classified into three tiers as described below.

### First Tier: Wireless Body Sensors (WBS)

In order to form a wireless body sensor network, it is proposed that each user should wear a number of wireless body sensors that are strategically placed on his/her body. The primary functions of these sensor nodes are to unobtrusively sample vital signs and transfer the relevant data to a personal server through wireless personal network implemented using ZigBee (IEEE 802.15.4). The biomedical sensors fitted on the body are supposed to measure blood pressure, heart beats, functioning of lungs, liver *etc.* Accordingly, various sensors such as ECG, accelerometer, oximeter, blood pressure monitor are fitted on a wearable suit to monitor a person from time to time. The sensor information is collected and transferred wirelessly to a smart sensor (gateway) that analyzes the ECG and other sensor data locally. The smart sensor processes the sensor data and monitors the patient's well-being, and in case of an emergency, it automatically generates alarms and forwards the analyzed data to the nodes present in the second tier through a mini-gateway present in the first tier. Ultimately, the information is passed to the emergency service provider (ESP) via internet as shown in Figure 1.

### Second Tier: Network Connectors (NC)

In an indoor environment, the network connector is considered as a sensor with higher computation and processing capacity and is mains powered. The devices of second tier collect data from the body sensors through a mini-gateway fitted in the smart suit as shown in Figure 2. The network connector (NC) transfers physiological data of a patient to the nearest gateway with help of other network connectors in a multi-hop fashion. In the second tier, the network connector (NC) is supposed to perform the following tasks:

- Initialization, configuration, and synchronization of WBSN nodes.
- Control and monitor operation of WBSN nodes.
- Collection of sensor readings from physiological sensors.
- Processing and integration of data from various physiological sensors providing better insight into the users state.
- Providing an audio and graphical user-interface that can be used to relay early warnings or guidance.
- Secure communication with remote healthcare provider via gateway nodes.

### Third Tier: Base Station (BS)

The third tier of the WBSN is composed of several base stations. Those base stations are powerful sensors with longer communication range and higher processing and battery power. They are mains powered and are connected to the internet to transmit data to the emergency service provider. It is assumed that a base station may send data to the internet directly or with help of other base stations in a multi-hop fashion. Accordingly, as shown in Figure 1, there are several base stations that are connected to the internet.

It is to be noted that the whole WBSN is composed of nodes from those three different tiers. Though, emergency service provider (ESP), doctors, hospitals are also part of the WBSN, our security mechanisms are limited with the nodes of those three tiers as discussed above. We do not consider the security issues of ESP as it could be a database server with different security mechanism.

## 4. System Model

In this section, we discuss the technical feasibilities of the devices that are to be used in three different tiers of the body sensor networks. Hence, a three tiered network model of the wireless body sensor networks is designed to construct a hierarchical key sharing mechanism for the data confidentiality and user authentication. Though dedicated secure micro-controllers may guarantee the integrity of the each node, we feel that such an architecture is too restrictive and cannot generalize to the majority of sensor networks. Besides, individual sensors after deployment could not be trusted. Hence, in order to provide stronger security methods, we classify the nodes into three different tiers based on the proposed network architecture. First, we analyze technical feasibilities of the sensors from the implementation point of view that are currently available in the market.

It is to be noted that the system architecture of wireless body sensor networks for mHealth applications could be either static or mobile wireless sensors. If a person wearing biomedical suit stays at home, the system architecture of wireless body sensor networks is considered as static as the body sensors are fixed within the suit and mobility of a patient is limited within the communication range of a network connector (NC) fixed in each home. The system architecture of such static biomedical wireless sensor networks is shown in Figure 2. When a person is at home, it is assumed that person having health problem wears a smart suit containing several body sensors. The suit itself contains several wireless sensors to monitor different health parameters such as heart, liver *etc.*, and the in-built sensors are connected wirelessly with each other. Besides, one of the sensors that is termed as a smart one acts as the mini-gateway, which collects data, aggregates them, analyzes the vital signs of the person and transmits data to the network connector located in the same house, if any life threatening situation arises. With help of the network connector, the data can be transmitted to the internet with help of the base stations. It is to be noted that the network connector is a smart device having interface to collect data from the wireless sensors using IEEE 802.15.4 MAC. Besides, it has interface to transmit data to the base station using the same medium access control protocol. Irrespective of the nature of the networks (either static or mobile), the wireless body sensor networks comprise of individual health monitoring systems that connect through the Internet to a medical server.

### 4.1. Technical Feasibilities

As we know, sensor nodes have very limited computational and power resources for executing several arithmetic and logical operations. Due to such hardware constraints of the sensor nodes, the public key certificates in asymmetric cryptographic algorithms like RSA [33] and Diff e–Hellman [34] are not suitable for WBSNs as the working memory of a sensor node is insufficient even to hold the variables. The symmetric algorithms, like AES and integrity/authentication algorithms like HMACs [35] incur high computational energy costs and are designed for the powerful workstations. Hence, the core asymmetric, symmetric and authentication algorithms in their current form are not suitable for the WBSNs as the computational cost is an overhead to the power consumption.

Based on different hardware constraints and the applications of WBSNs, we have classified the sensor nodes into three categories such as the generic wireless body sensors, special-purpose network connectors and the high-bandwidth base stations. The hardware specifications of these nodes are given in Tables 1, 2 and 3, respectively. As given in Table 1, Telos-B Mote [36] TPR2420 is developed by Crossbow and is an open-source platform. TPR2420 combines all essentials for lab studies into a single platform including USB programming capability, an IEEE 802.15.4 radio with integrated antenna, a low-power MCU with extended memory and an optional sensor suite. Besides, it includes several other features like IEEE 802.15.4 compliant RF transceiver, a globally compatible ISM band 2.4 to 2.4835 GHz, 250 kbps data rate, integrated onboard antenna, 8 MHz TI MSP430 microcontroller with 10 kB RAM, low current consumption, 1 MB external flash for data logging, programming and data collection via USB and runs TinyOS 1.1.11 or higher. More technical specification [36] of this generic sensor is given in Table 1.

The Rockwell WINS Hidra nodes consist of 2 × 2 boards in a 3.5 × 3.5 × 3 enclosure. There are various sensors such as seismic (geophone), acoustic, magnetometer, accelerometer, temperature, pressure and uses RF communications. Technical specification [37] of this sensor is given in Table 2.

The Stargate NetBridge [38] is an embedded sensor network gateway device, which can act as a base station to send and receive data with other base stations. It can connect to other sensor nodes to an existing Ethernet network. It is based on the Intel IXP420 XScale processor running at 266 MHz. It features one wired Ethernet and two USB 2.0 ports. The device is further equipped with 8 MB of program FLASH, 32 MB of RAM and a 2 GB USB 2.0 system disk. Stargate NetBridge also contains a built-in Web server and sensor network management tool. The network management tool can automatically identify what types of sensor boards are plugged into the nodes of the wireless sensor network and will instruct Mote Explorer to display the data accordingly. It is truly plug-and-play with minimal overhead for configuration and administration. Technical specification [38] of this node is given in Table 3.

**Figure 3.**
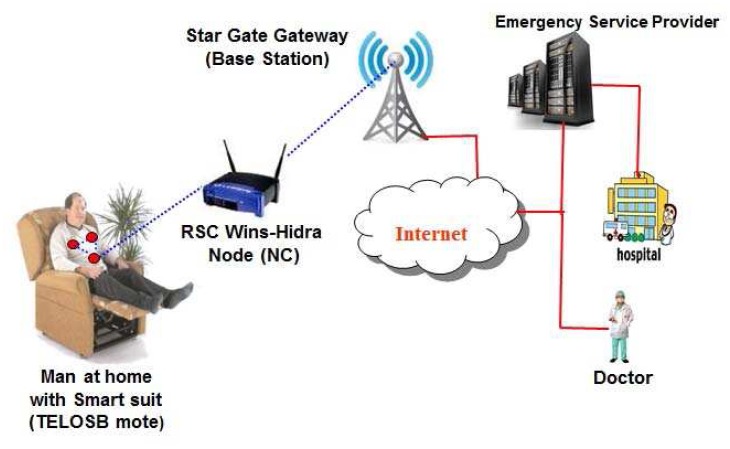
The indoor mHealth with different types of nodes that form the WBSN.

As shown in Figure 3, we propose to use the generic TelosB Mote-TPR2420 as the body sensors, RSC Wins-Hidra nodes as the special purpose network connectors and Stargate NetBridge as the base station, which is connected to the internet. In Figure 3, though the wireless body sensor network is represented by a single node, several nodes are present in the network in a realtime application environment. Classifications of nodes based on the proposed network architecture is described in the following subsection.

### 4.2. Classification of Nodes

**Figure 4.**
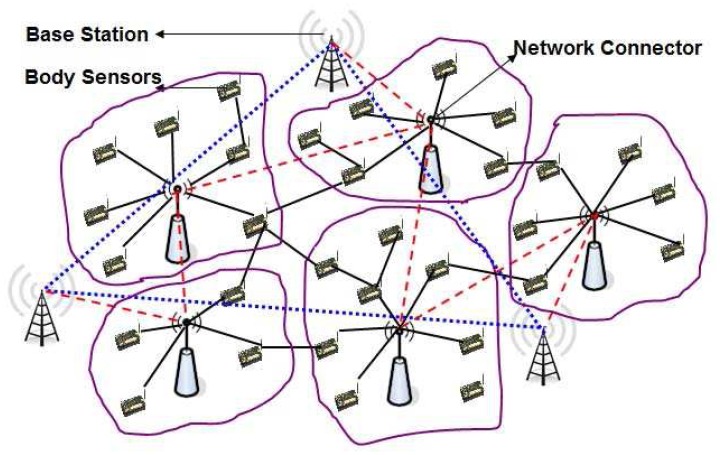
Logical view of the three tiered network architecture of WBSNs.

In order to design a lightweight security mechanism for the WBSN, we classify the whole wireless body sensor networks into three types of nodes. They are the Sensor nodes (SN), Network Connectors (NC) and Base stations (BS) as shown in Figure 4. The whole network is divided into several clusters based on standard clustering algorithms [39,40], where each cluster has only one cluster head. The body sensors (SNs) and network connectors (NCs) are deployed randomly, whereas multiple base stations are deployed manually depending on the location and communication range of the network connectors. As soon as SNs and NCs are deployed, they organize among themselves and form the cluster based on different criteria such as their location from the sensors, communication range and limitation of the number of sensors to be connected with them. We assume that several protocols [39,40] are already designed to handle efficient clustering methods. In our protocol, each cluster head is assumed to be a network connector (NC) that controls several SNs, which is quite reasonable as per the existing clustering algorithms. The NCs of different clusters exchange their collected data with each other. Besides, the NCs also transmit their data to the nearby base station (BS) and finally to the user or network manager, which is located somewhere far away from the monitoring region that access the sensed data and monitors the network via the base stations (BSs). The nodes of the three tier network architecture can be designed as given below.

**Tier 1 Nodes:** The first tier nodes of the network model are the set of generic sensor nodes (SN) like Telos-B Motes [36], which are put in a wearable smart suit. Their functions are simple, specific and are usually operated independently. They sense the medium, collect the raw data and forward it to the next hop neighbor nodes, which are ultimately forwarded to the nodes of the second tier. The hardware specifications of such nodes are shown in Table 1.
**Tier 2 Nodes:** Some special purpose sensor nodes like RSC Wins-Hidra Nodes [37], limited number of which are deployed over the monitoring region. In each cluster, there exists only one network connector (NC), which is cluster head of that cluster, and receives data from the SNs of its own cluster and forwards them to the NCs of other clusters. These nodes are assumed to be more powerful in terms of computation with longer battery life and larger memory than the SNs. The technical specification of these nodes is presented in Table 2. Each NC of the network has a unique ID, which may be assigned based on its cluster ID. NCs can track events or targets using the sensors of its own cluster and prepare the final report using data fusion and aggregation techniques to forward the final data to the nodes of the third tier. It is to be noted that each logical cluster can be considered as one home of a patient, where wearable wireless body sensors are used.
**Tier 3 Nodes:** The high-bandwidth sensing and communicating nodes like Stargate gateways [38] form the third tier of the network and are known as the BS of the WBSNs. As per our proposed network architecture, we consider multiple base stations, whose operating characteristics are given in Table 3. These base stations are low-Power, small-Size, 400 MHz, Linux single board computer with enhanced communications and sensor signal processing capabilities. These Stargate gateways use Intel's latest generation 400 MHz X-Scale processor. In addition to traditional single board computer applications, the Stargate directly supports applications designed around Intel's Open-Source Robotics initiative as well as TinyOS-based Wireless Sensor Networks. These nodes have relatively powerful processing, memory, and transmission capacity and have long battery life, and they could be mains powered so that there is no power constraint. It is assumed that these base stations (BSs) are connected wirelessly to the user or network manager via internet and satellite.

## 5. Security Architecture

In this section, we propose our data confidentiality and user authentication algorithms. In the proposed schemes, three types of keys are used for necessary security verifications and data confidentiality. It is to be noted that our proposed algorithms for data confidentiality and user authentication are applicable to all types of nodes irrespective of its presence in any particular tier.

### 5.1. Key Arrangements

**Figure 5.**
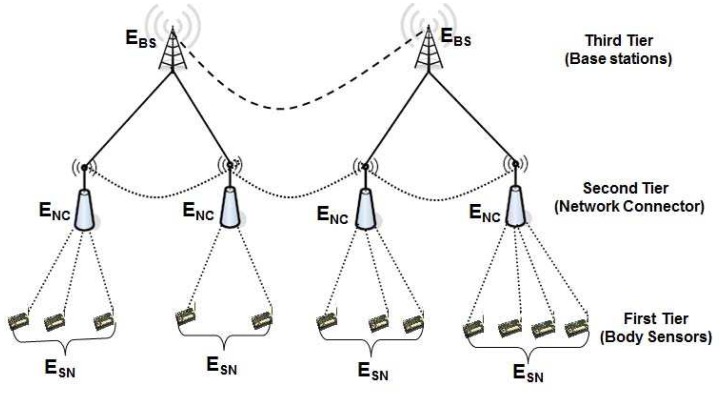
The hierarchical key sharing architecture of the whole network.

It is assumed that communication among SNs in each clusters (e.g., nodes of the first tier) is only broadcasting and routing of packets among NCs and NCs to BSs (e.g., communication among nodes of second tier and second with third tier) is only unicasting by nature. We propose three different types of keys for the whole network, which is summarized below and hierarchical key sharing architecture is shown in Figure 5.

**Sensor Key:** Each sensor of the network has a unique secret key, which is common for all the sensors of the network irrespective of its location in any cluster. This common secret key is denoted as *E_SN_*. We assume that the SNs of each clusters are fixed with respect to their cluster head (NC).
**Network Connector Key:** Each cluster head that is otherwise known as NC has a unique secret key, which is denoted as *E_NC_*. The network connector key *E_NC_* of a NC (cluster head) is same for all the NCs of the network. It is to be noted that the network connector key *E_NC_* is distinct from the sensor key *E_SN_*. In order to make necessary security verification with the sensors, it stores *E_SN_* and also stores its own key *E_NC_* for making necessary security verifications with other NCs of the network.
**Base Station Key:** Each BS of the network has a unique secret key, which is common for all BSs and is denoted as *E_BS_*. This key is distinct from the *E_SN_* and *E_NC_*. Each BS stores *E_BS_* and *E_NC_*, in order to make necessary security verifications with other BSs and NCs, respectively.

As shown in Figure 5, the sensors that belong to the first tier of the network have only one secret key *E_SN_*, which is same for all the nodes of the first tier. The NCs that belong to the second tier of the network have only one secret key *E_NC_*, which is same for all the nodes of the second tier. Similarly, the BSs that belong to the third tier of the network have only one secret key *E_BS_*, which is same for all the nodes of the third tier. It is proposed that SNs of each cluster store only one key *E_SN_* for necessary security verifications with SNs of the same or other clusters. Each NC stores *E_SN_* and *E_NC_* for necessary security verifications with SNs of its own cluster and NCs of other cluster, respectively. Similarly, each BS stores *E_NC_* and *E_BS_* for necessary security verifications with NC and other BSs, respectively.

We consider the upstream data flow from the SNs to the NCs, which is reasonable as sensors are meant to probe the environment to detect a target or event and inform it to the base station through gateway nodes. Hence, each NC uses *E_SN_* to maintain the data confidentiality with the sensor nodes it is attached to. Under special circumstances, if a NC issues mission, sends queries and interests, to the SNs of its cluster, it uses its own key *E_NC_* to verify the data confidentiality received from the sensors. It is assumed that the secret keys of SNs, NCs and BSs are assigned at the manufacturing stage as the key assignment at the network formation phase in a hostile medium is not secure. It is to be noted that since each SNs, NCs or BSs has the same secret key, key assignment and management in the post deployment of nodes does not require any computation and key establishment. For example, sensor nodes *A, B* and *C* can use their common secret key whether they are in the same or in different clusters after the deployment of the nodes as the common keys of all sensors are same.

### 5.2. Data Confidentiality

Before we describe the data confidentiality algorithms, we present here some of the useful terms that we have used in the next subsequent steps.

- Let, A and *B* be two sensors present in the first tier of the network, which transmit *x* -bits of message *M* (plain text).
- *n* is a pre-assigned integer such that *0* < *n*^2^ < *x*. It is known to both the sender and the receiver in advance, e.g., during the network construction phase and the value is fixed for all the messages.
- Divide the whole message *M* into *k*-numbers of sub-messages *M*_1_, *M*_2_,…, *M_k_* of *n*^2^-bits each such that *M*_1_ *| M*_2_ | … | *M_k_* denotes the concatenation of *k*-numbers of message into *M*, where $k=\left\lceil \frac{x}{n^{2}}\right\rceil .$
- *E_SN_* is the secret key of *n*^2^-bits, which is common for sensor nodes *A* and *B* and ⊙ is a user defined binary operation. Both *E_SN_* and ⊙ are known to both sensors a priori. However, ⊙ acts like a session key and types of binary operations can be changed from time to time by the cluster head GN to enhance the security.
- Using the secret key *E_SN_*, let the cipher text {*M*}*_ESN_* be generated from the plain text *M*.

Suppose the plain text message *M* of *x*-bits is sent by any sensor to another one after encrypting it by the *n*^2^-bits secret key *E_SN_*. Then the encrypted message is:

$${\left\{M\right\}}_{E_{SN}}=M*E_{SN}(\text{mod p})$$

where *p* is a prime number of order 512 bits in the Galois field. Though *E_SN_* is the common secret key among the sensors and is only known to the sender and receiver, it could be possible that the adversary can capture any packet sent to the destination to intercept the cipher message and may hack the common secret key. Once adversary analyzes the cipher message and gets the secret key from any of the sent packet, it will be easier for it to break the confidentiality of subsequent packets. Thus the security of the whole network is compromised. In order to overcome such problem, we propose a stronger confidentiality scheme. Instead of sending any single key in a single data packet, new encrypted keys are generated based on some physical situations, such as time of sent message or local temperature at the time when the message is sent *etc.*

Based on the proposed physical situation based key generation mechanism, let us assume that a time stamp matrix *T_i_* of *n*^2^-bits, for ∀*i* = 1, 2, 3,…, *k*, is considered. For each message, sent at different instant of time, different time stamp matrices *T_i_* are generated by using the common secret key *E_SN_*. Thus, several new secret keys *E_i_^SN^* are generated, which are only known to the sender. The subsequent new secret keys can be generated as follows:

$$\begin{array}{c} {E_{1}}^{SN}=E_{SN}⊙T_{1}\\ {E_{2}}^{SN}=E_{SN}⊙T_{2}\\ {E_{3}}^{SN}=E_{SN}⊙T_{3}\\ \vdots \\ {E_{k}}^{SN}=E_{SN}⊙T_{k} \end{array}$$

where, *T*_1_, *T*_2_, *T*_3_,…, *T_k_* are the time stamp matrices, which are based on the local time at which a message is sent and ⊙ is a user defined binary operation that is only known to the sender and receiver. It is to be noted that ⊙ acts like a session key among the SNs as well as the SNs with NCs. Besides, NC of different clusters may have different types of binary operations, which could be changed from time to time.

Now break the original message *M* into *k*-number of messages *M*_1_, *M*_2_,…, *M_k_*, each of *n*^2^-bits. Since $k=\left\lceil \frac{x}{n^{2}}\right\rceil$, it is obvious that for $\frac{x}{n^{2}}$ is not a whole number, *M* can be broken into (*k* − 1) number of messages of *n*^2^-bits each, and another one message (*M_k_*) of [*x* − *n^2^* * (*k* − 1)]-bits, which is less than *n*^2^-bits. In this case, the last message *M_k_* will have [*x* − *n*^2^ * (*k* − 1)]-bits of message and rest bits are garbages such as ♡ or anything else. However, if $\frac{x}{n^{2}}$ is a whole number, *M* is broken into *k*-numbers of messages, each having *n*^2^-bits. The new cipher message *C_i_* is generated by taking the messages *M_i_* and the new encrypted matrix *E_i_^SN^*. Thus the transmitted cipher message at different instants *T*_1_, *T*_2_, *T*_3_,…, *T_k_* are:

$$\begin{array}{c} C_{1}=M_{1}*{E_{1}}^{SN}(\mathrm{mod}\text{p})\\ C_{2}=M_{2}*{E_{2}}^{SN}(\mathrm{mod}\text{p})\\ \vdots \\ C_{k}=M_{k}*{E_{i}}^{SN}(\mathrm{mod}\text{p}) \end{array}$$

Finally, the sender transmits the original message *M* in form of the cipher messages *C_i_*. The data packet transmitted by the sender contains *n*^2^-bits of the cipher message and *n*^2^-bits of time-stamp matrix and the whole message *M* is transmitted for *k*-times. Upon receiving the data packets, the gateway nodes decrypt each cipher messages using the secret key *E_SN_*and the time-stamp matrix *T_i_*. Since the secret key *E_SN_* and binary operation ⊙ are only known to the sender and the receiver, the data confidentiality cannot be lost even though adversary is able to hack the cipher message. Thus, similar procedures of confidentiality schemes can be applied for establishing the necessary data confidentiality between the SNs and NCs. However, when the NC receives the data packet from any SN, it uses its secret key *E_SN_* instead of *E_NC_* to decrypt the message. But, if any message is sent from one NC to other, it uses *E_NC_* to encrypt it before sending to other NC of the network.

### 5.3. Authentication

In this subsection, a low complexity authentication scheme is proposed, which is applicable to the nodes of all three tiers of the network. Each cluster head NC assigns a unique ID to each of the sensors present in its cluster. Similarly, each NC has a unique ID such as the ID of the cluster that it belongs to and each BS has also a unique ID too. Each NC stores ID of the sensors that belong to its own cluster and ID of other NCs too. Similarly, each BS stores ID of other BSs and ID of NCs that are connected to it. It is to be noted that the number of NCs and BSs deployed over the network is very small as compared to the number sensors (SNs). Besides, their storage, power and computational capabilities are higher than the SNs. Hence, it is reasonable to assume the maintenance of ID of the SNs by the NC of a particular cluster. The unique ID of the SN, NC or BS is considered as the public key for the authentication purpose, which is described as follows.

Let,
*y*:ID of the SNs/NCs/BS be the public key.*m*:The cipher message, encrypted as per the data confidentiality technique described in the previous section.*a, b*:Unknown variables*x*:Sender's private key

Now the sent message from *A* to *B* is:

- *A → B: A*(*y*, *a*, *b*, m) and the cryptographic function is:
- *x*^2^ = y (mod n) such that

$$\begin{array}{l} a-b\equiv (m+1)*\frac{x}{\alpha }(\mathrm{mod}\text{n})\\ a+b\equiv (m^{2}-m+1)*x\alpha (\mathrm{mod}\text{n}) \end{array}$$

where, *α* is a random number and *n* is a 1,024 bits composite number. Upon receiving the packet containing *y*, *a*, *b* and *m*, receiver *B* can calculate *a*^2^ **−** *b*^2^ ≡ (*m*^3^ + 1) * *y* (mod n).

Ultimately, node *B* uses the public key cryptographic mechanism to calculate the value of *n*. If it matches with its preserved value of *n* with *A*'s value of *n*, then it authenticate *A* as a legitimate sender. It is to be noted that *y* is the ID of the sender and for each sender there will be a unique *n* that should match with the receiver's *n*.

### 5.4. Theoretical Analysis

In this section we analyze the computational and storage cost of our protocol due to the key updating, establishment, encryption and decryption operations during the confidentiality verification. It is to be repeated here that in our protocol, we do not need any key updating mechanism as we assign a single key to all the sensors, another single key to all NCs, and also a single shared key to all the BSs of the network. Hence, in our protocol, there is no computational cost required in establishing the keying relationship among either the SNs or NCs or BSs. Also, our protocols do not impose any computational burden for key updating or in establishing the keying relationship. However, the computational cost in encrypting or decrypting the message can be calculated as follows:

- In case of SNs: Suppose, in a cluster a node has *n* different neighbors and *x_i_*, *i* = 1, 2, 3,…, *n* be the number of neighbors of those ***n*** nodes. Hence, total number of required encryptions is: *E_T_* = Σ*x_i_*, for *i* = 1, 2, 3,…, *n*. Similarly total number of decryption is also *D_T_* = Σ*x_i_*, for *i* = 1, 2, 3,…, *n*.
  In a cluster, average number of symmetric operations are $=\frac{2\sum x_{i}}{\left(n+\sum x_{i}+1\right)}$
- In our protocol NCs communicate with other NCs by unicasting the message. Suppose, the whole network has *m* numbers of NCs. In the worst case, an NC will have at most (*m* − 1) neighbors. The average number of encryptions and decryptions in case of the NCs is $=\frac{2(m-1)}{m}$
- In case of BSs: Suppose, the whole network contains *p* number of BSs. As the communication among the BSs is also unicasting, average number of encryptions and decryptions is $=\frac{2(p-1)}{p}.$

In our protocol, *p* < *m* < *n*. Hence, total average number of encryption and decryptions operations $=\frac{2\sum x_{i}}{\left(n+\sum x_{i}+1\right)}+\frac{2(m-1)}{m}+\frac{2(p-1)}{p}.$. Besides, in our protocol, a node stores only two types of keys, e.g., *E_SN_* and *E_NC_* and keys are same for all the nodes the cluster. Hence, there is no requirement to store the chain of keys for its neighbors. If *l*_1_ is the key length of *E_SN_* and *l*_2_ is the key length of *E_NC_*, then the total key length is required to store in each SN is *l = l*_1_ + *l*_2_. Though memory space is the scarce resource for the sensor nodes, for a reasonable key length of *E_SN_* and *E_NC_*, storage is not an issue in our protocol. It is observed that the storage requirement, encryption and decryption computational costs of our protocol is least, which is evidenced from the simulation results as given in the following section.

## 6. Performance Evaluation

In this section, we evaluate performance of our proposed schemes through simulations and compare the performances in terms energy consumption, computation and storage.

### 6.1. Simulation Setup

Our proposed data confidentiality and authentication schemes are evaluated using ns 2.33. A rectangular monitoring region of size 100 x 100 m^2^ is taken and 1,000 nodes are deployed over it. Based on our proposed scheme, total number of deployed nodes include three different types of nodes such as Sensor nodes (SN), Network Connectors (NC), and Base station (BS). Though SNs and NCs are deployed randomly, BSs are deployed manually. The schemes are evaluated with variable node numbers of 100 through 1,000. In each case, 90% of the total deployed nodes are taken to be SNs, 9% of them are NCs and only 1% are BSs. In our simulation, IEEE 802.15.4 MAC is considered as the medium access mechanism during the communication between sensors and sensors with gateways, whereas unicasting is used for the communication between BSs and BS with NCs. The communication range of each node is fixed at 10 meters. The energy consumption due to transmission of a packet by sensors or gateway nodes is considered to be 0.2 joules. It is to be noted that our schemes consider only upstream communication, *i.e.*, only sensors transmit data to their neighbor sensors or to its cluster head as the gateway.

### 6.2. Simulation Results

**Figure 6.**
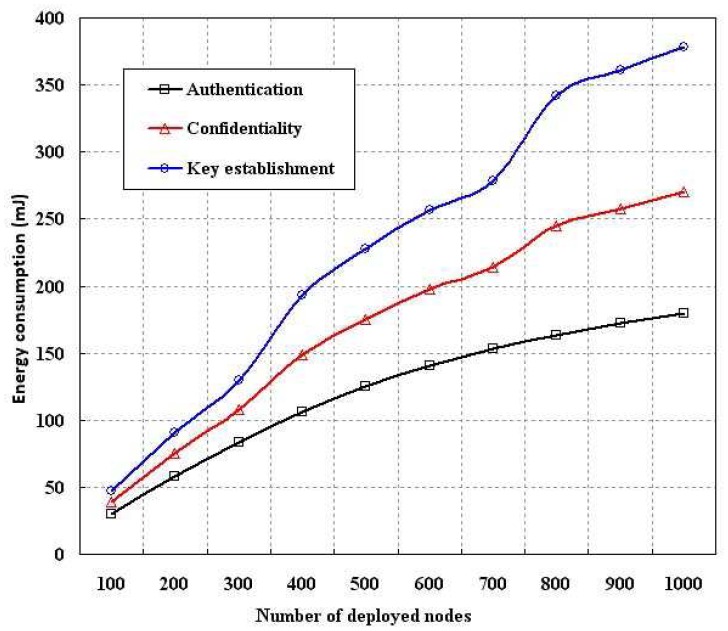
Evaluation of energy consumption with different number of nodes.

**Figure 7.**
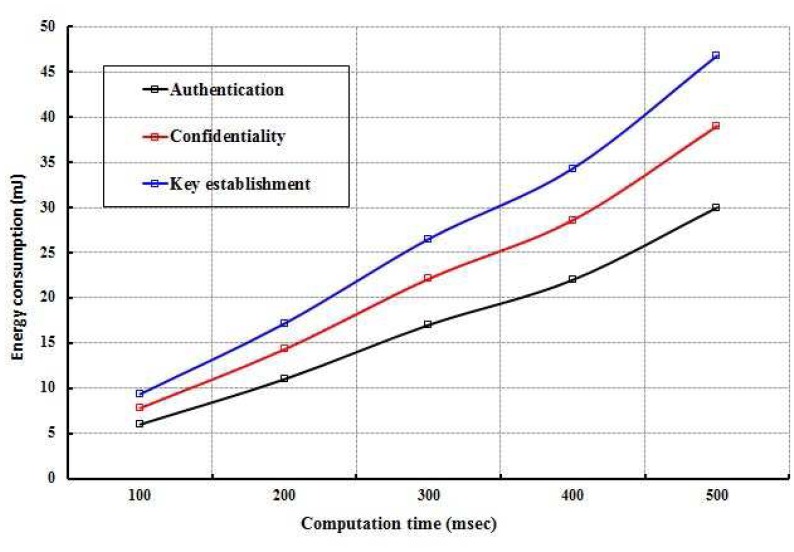
Evaluation of energy consumption with computation time.

**Figure 8.**
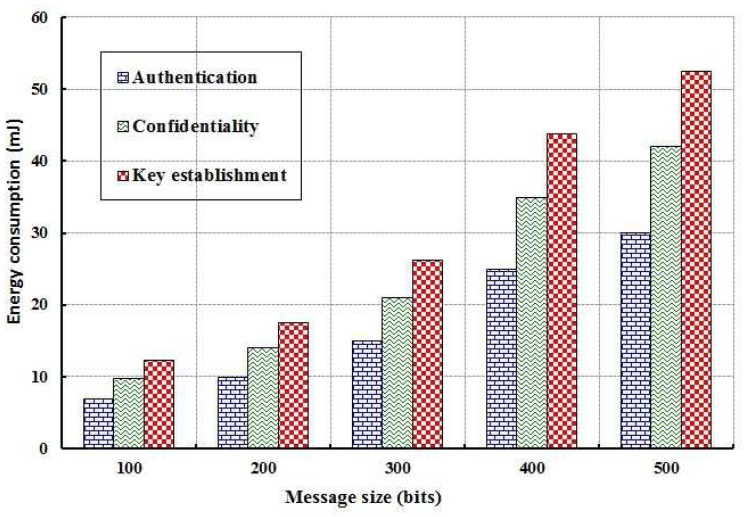
Evaluation of energy consumption with different message sizes in bits.

In order to evaluate the performance of proposed security schemes, we simulated the energy consumption, computation time, memory usage and control packets overhead as compared to standard key establishment schemes with our authentication and confidentiality schemes. As per our proposed scheme, each sensor is given only one key (*E_SN_*) as contrary to the standard key establishment protocols. The energy consumption is evaluated as shown in Figures 6, 7 and 8 in terms of different number of nodes, computation time and size of the transmitted packets. In our simulation, energy consumption is defined as the amount of energy consumed in completing a process such as establishing the keys among the deployed sensors or authenticating a node based on our scheme or maintaining confidentiality as per our model. As shown in Figure 6, it is found that energy consumption for establishing the keys among different number of nodes is higher than the confidentiality and authentication. However, in each case energy consumption increases if number of deployed nodes are increased. Energy consumption in authentication is less than the energy consumption due to confidentiality implementation.

**Figure 9.**
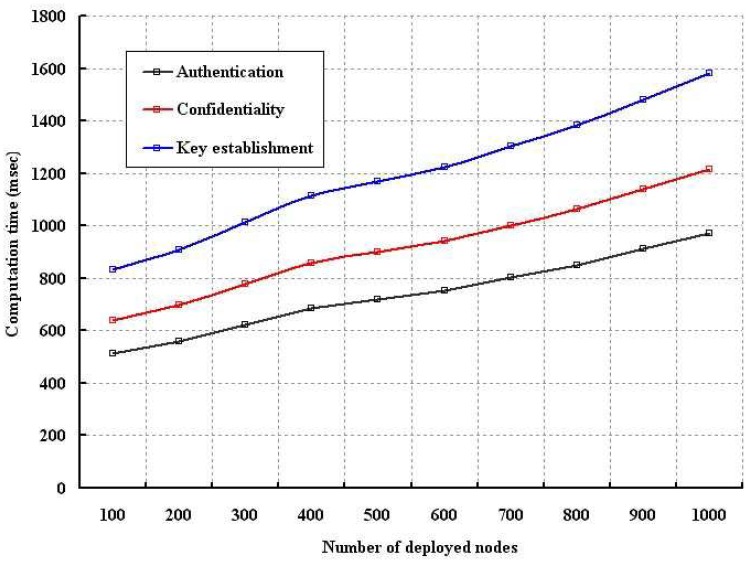
Evaluation of computation time with different number of nodes.

As shown in Figure 7, energy consumption of the whole network is evaluated for different values of computation time in establishing the keying relationship among the nodes and in executing the data confidentiality and authentication mechanisms. It is observed that energy consumption is directly proportional to the computation time. Obviously, energy consumption of the network is increased due to longer computation time. Energy consumption of the nodes is also evaluated with size of the packets that are exchanged between the nodes in executing the proposed security mechanisms as shown in Figure 9. It is found that energy consumption is more in case of key establishment as compared to the authentication and data confidentiality. Least amount of energy is consumed in authentication as very few messages are exchanged and each packet is segmented and therefore requires limited energy to transmit them.

**Figure 10.**
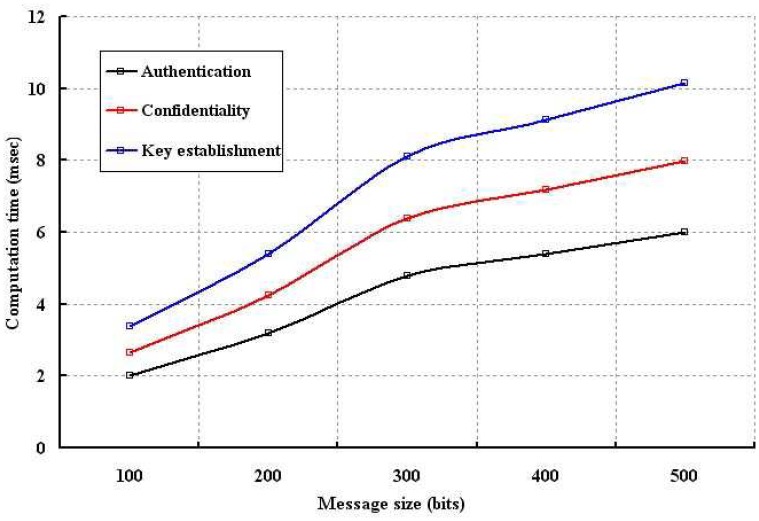
Evaluation of computation time with different packet size in bits.

The computation time is analyzed for different number of nodes and size of the packets as shown in Figures 9 and 10, respectively. In our simulation, computation time is defined as the time require to authenticate a node by the sensors, network connector or base station and also the time taken to measure the confidentiality of a sent packet. As shown in Figure 9, our data confidentiality and authentication schemes are simulated with different number of nodes. It is found that the computation time is longer in case of key establishment as compared to our proposed security schemes. Though implementation of our confidentiality mechanism requires more computation time, it is visibly shorter than the key establishment scheme. Our authentication scheme takes least computation time. Moreover, with smaller computation time, our scheme can provide better protection to the wireless body sensor networks. As shown in Figure 10, it is observed that confidentiality or authentication requires longer computation time if size of the sent packet is large. As per our scheme, confidentiality requires more computation time as a single packet is segmented into several packets. However, our security schemes in form of authentication and confidentiality outperforms over key establishment.

**Figure 11.**
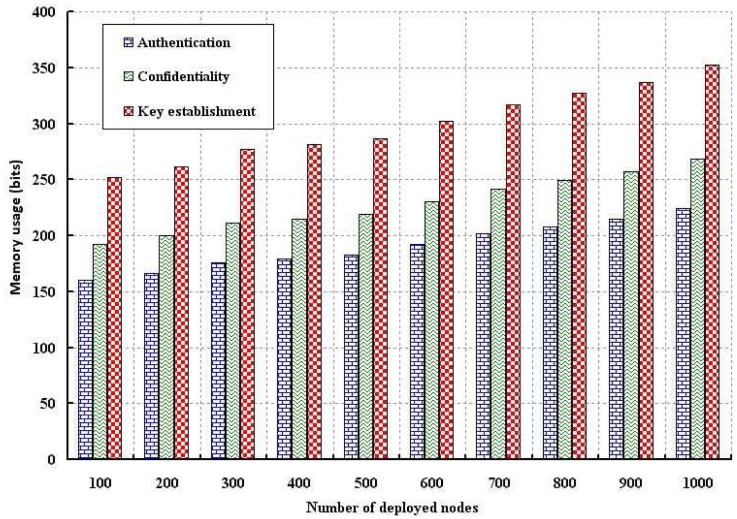
Evaluation of memory usage with different number of nodes.

**Figure 12.**
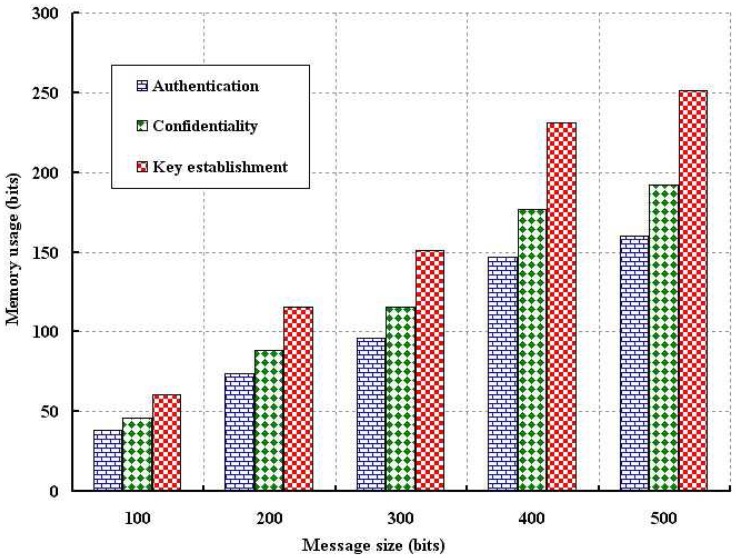
Evaluation of memory usage with different packet size in bits.

The memory usage due to authentication and confidentiality is simulated and the result is compared with the key establishment scheme, as shown in Figures 11 and 12. The memory usage is defined as the amount of memory require to store the data such as sensor (*E_SN_*), network connector (*E_NC_*) and base station (*E_BS_*) keys and necessary computation parameters. As shown in Figure 11, memory usage is analyzed with different number of nodes. Since we use very limited parameters to verify authentication and confidentiality, memory usage is smaller as compared to the key establishment scheme. Of course, more memory storage is required if number of nodes are deployed as we consider the average memory usage taking all deployed nodes. As shown in Figure 12, memory usage is analyzed with size of the data packet. It is observed that our schemes require less storage as compared to key establishment protocol.

**Figure 13.**
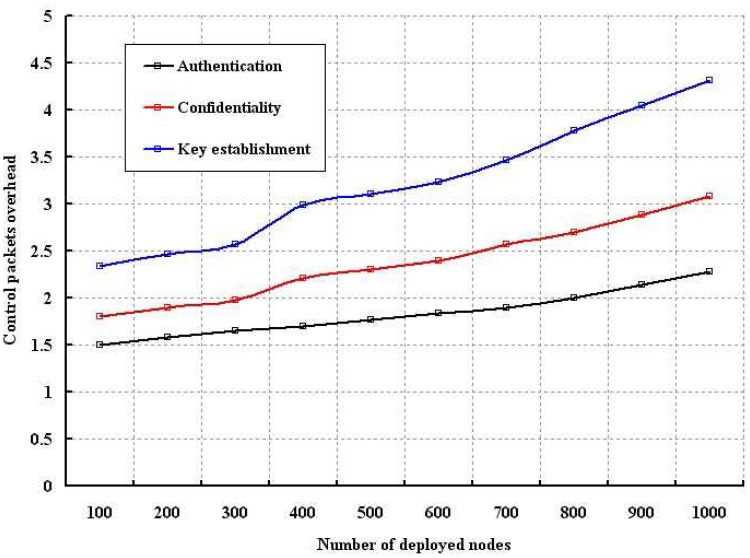
Control packet overhead with different number of nodes.

The packet overhead in establishing the key and communication during our proposed authentication and confidentiality schemes is simulated with number of nodes and the result is shown in Figure 13. Though the key establishment needs less number of control packets for smaller number of nodes, it gradually increases with higher number of nodes. Besides, our authentication and confidentiality schemes use less number of control packets as inferred from Figure 13.

## 7. Conclusions

Different researchers propose different security schemes for the wireless sensor networks. However, very few research works are there on security mechanisms of WBSN. Hence, the main motivation to design security protocols for the WBSNs should be light weight in terms of computation and memory storage so that less amount of energy should be consumed in implementing them. Besides, number of keys distributed among nodes for the necessary security verification should be limited. In order to minimize the number of shared keys, we propose here a three tiered network architecture for the WBSN for realization of an mHealth application and design light weight, low complexity data confidential and user authentication schemes for the WBSNs. In stead of using several keys to be stored with each node, we propose a segmented based data packet and communication paradigm, where secret keys are embedded within those segmented packets itself. According to our scheme, a sensor stores only one key, a network connector or a base station stores only two keys. We propose to use only three different types of keys for the three tier network model, which can provide security as well as can minimize the memory usage, computation time and energy consumption. From the evaluation of our schemes, it is found that our protocol is suitable for the wireless body sensor networks within its current physical constraints. Our proposed schemes can be applicable for the WBSNs in medical applications such as mHealth.

## Figures and Tables

**Table 1.** Prototype of generic sensor nodes (TelosB Mote-TPR2420).

**Features**	**Specifications**
Processor	8 MHz MSP430F1611 microcontroller
Processor Performance	16-bit RISC
Memory	10 KB RAM and 48 KB Flash Memory
Measurement Serial Flash	1,024 K bytes
RAM	10 K bytes
Configuration EEPROM	16 K bytes
Radio	Integrated onboard antenna
Analog to Digital Converter	12 bit ADC
Digital to Analog Converter	12 bit DAC
Data Rate	250 kbps
MAC Protocol	IEEE 802.15.4 compliant
Transceiver	UART(Universal Asynchronous Receiver Transmitter)
Frequency band	2,400 MHz to 2,483.5 MHz
Indoor Range	20 m to 30 m

**Table 2.** Prototype of special purpose network connectors (RSC Wins-Hidra Nodes).

**Features**	**Specifications**
Processor	Intel StrongARM 1100@133 MHz, 150 MIPS
Memory	1 MB SRAM, 4 MB Flash memory
Radio	RDSSS9M Radio @ 100 kbps, 1–100 mW, 40 channels
Data Rate	100 kbps
MAC Protocol	IEEE 802.15.4 compliant
Transceiver	RF communications, TDMA MAC with multihop routing

**Table 3.** Prototype of high bandwidth base stations Stargate NetBridge.

**Features**	**Specifications**
Processor	IXP420 @ 266 MHz
Memory	8 MB Program FLASH memory
RAM	32 MB
USB FLASH Disk	2 GB
Input and output	1 × RJ45 Ethernet (IEEE 802.3, 802.3u)
